# Supplementary material for: Occupational injury prevalence and predictors among small-scale sawmill workers in the Sokoban Wood Village, Kumasi, Ghana
Source: PLoS One. 2024 Apr 10;19(4):e0298954. doi: 10.1371/journal.pone.0298954 (PMC11006181; doi:10.1371/journal.pone.0298954)
Supplement: S1 Table — (DOCX) [file pone.0298954.s001.docx]

**S1 Small-scale sawmill companies and their required sample size**

| **Sawmill companies** | **Total population** | **Required sample size** |
| --- | --- | --- |
| 1 | 29 | 22 |
| 2 | 38 | 29 |
| 3 | 49 | 37 |
| 4 | 17 | 13 |
| 5 | 28 | 21 |
| 6 | 20 | 15 |
