# Supplementary material for: Occupational injury prevalence and predictors among small-scale sawmill workers in the Sokoban Wood Village, Kumasi, Ghana
Source: PLoS One. 2024 Apr 10;19(4):e0298954. doi: 10.1371/journal.pone.0298954 (PMC11006181; doi:10.1371/journal.pone.0298954)
Supplement: S2 Table — (DOCX) [file pone.0298954.s002.docx]

**S2 Table: Multi-collinearity test results**

| **Variables** | **VIF** | **1/VIF** | **R-Squared** |
| --- | --- | --- | --- |
| Education | 1.12 | 1.06 | 0.1095 |
| Cadre of staff | 1.22 | 1.10 | 0.1808 |
| Work hours per week | 1.10 | 1.05 | 0.0902 |
| Workspace design | 1.32 | 1.15 | 0.2407 |
| Lighting at the workplace | 1.14 | 1.07 | 0.1223 |
| Supplied PPE | 1.12 | 1.06 | 0.1032 |
| Income | 1.36 | 1.17 | 0.2632 |
| Work experience | 1.18 | 1.09 | 0.1536 |
| Mean VIF | 1.19 |  |  |
