# Supplementary material for: Occupational injury prevalence and predictors among small-scale sawmill workers in the Sokoban Wood Village, Kumasi, Ghana
Source: PLoS One. 2024 Apr 10;19(4):e0298954. doi: 10.1371/journal.pone.0298954 (PMC11006181; doi:10.1371/journal.pone.0298954)
Supplement: S1 File — (DOCX) [file pone.0298954.s003.docx]

**Questionnaire**

**SECTION A: BACKGROUND AND WORK-RELATED CHARACTERISTICS**

1. What is your age as at last birthday? …………
2. What is your gender? [ ] Male [ ] Female
3. What is your highest educational level?

[ ] No formal education [ ] Basic Education [ ] SHS [ ] Tertiary [ ] others, please specify……………...

1. Marital status [ ] Single [ ] Co- habitation [ ] Married [ ] Divorced [ ] Widowed [ ] others, please specify ………….
2. What is your monthly income? >500 [ ] 500 – 999 [ ] 1000+……………
3. How long have you been working here? …………
4. What role do you play here? [ ] Operator [ ] Engineer [ ] Driver [ ] Administrative worker [ ] Technician [ ] others, please specify ……………………………
5. How many hours do you work in the day? …………..
6. How many days do you work in a week? ……………
7. How often did you usually have any kind of drink containing alcohol during the last 2 years? Not at all [ ] [ ] Occasionally [ ] Every day [ ]
8. Are you supplied with personal protective equipment by your company? Yes [ ] No [ ]
9. If yes, how often do you use them at the workplace? Always [ ] Sometimes [ ]
10. How would you describe the lighting system at your workplace? Good [ ] Poor [ ]
11. How would you describe the workspace design at your workplace? Good [ ] Poor [ ]

**SECTION B: ACCIDENTS AND INJURIES ON THE JOB**

27. Have you ever suffered any kind of injuries in the last 12 months from today? [ ] No [ ] Yes

**If yes in the above, answer the following questions**

28. What caused the accident? [ ] moving machine part/sharp object [ ] Hit by an log/object [ ] vehicle [ ], lifting heavy objects [ ] others, please specify……………………………………

29. What kind of injury did you suffer? [ ] Burns [ ] Cuts [ ] Abrasions [ ] Internal injury [ ] others, please specify

30. Which part of your body has been injured whiles working here? [ ] Arm [ ] Leg [ ] hand [ ] foot [ ] Eyes [ ] Head [ ] Others, please specify ………………………

1. Did the injury require hospitalization? Yes [ ] No [ ]
2. If yes, how long were you hospitalized? …………
3. Were you given a compensation when you got injured? Yes [ ] No [ ]
4. How was the injury treated? Selfcare [ ] Received medical attention [ ] Other please specify…………
