# Supplementary material for: Occupational injury prevalence and predictors among small-scale sawmill workers in the Sokoban Wood Village, Kumasi, Ghana
Source: PLoS One. 2024 Apr 10;19(4):e0298954. doi: 10.1371/journal.pone.0298954 (PMC11006181; doi:10.1371/journal.pone.0298954)
Supplement: Attachment — Submitted filename: AUTHORS RESPONSE.docx [file pone.0298954.s006.docx]

27^th^ January 2024

The Editor-In-Chief

PLOS One

**AUTHORS’ RESPONSES TO REVIEWERS’ COMMENTS – OCCUPATIONAL INJURY PREVALENCE AND PREDICTORS AMONG SMALL-SCALE SAWMILL WORKERS IN THE SOKOBAN WOOD VILLAGE, KUMASI, GHANA**

**Reviewer’s comment:** Cochrane formula used must be referenced/source cited, first formula written must be given the equation number and reference/citation.

**Authors’ response:** This has been done as suggested.

*The Cochrane formula was used to estimate the sample size for this study [28]:*

$Sample size, SS= \frac{Z^{2}pq}{d^{2}}$*,	Equation 1 [28]*

**Reviewer’s comment:**  It’s not clear why adjusting for the 25% (quite high) non-response rate and go for the 138? Meanwhile, you targeted all staff 181 (census sample) and that should be enough to indicate and then report on the response/non-response rate.

**Authors’ response:** Please, we did not target all staff. The study population was rather limited to small-scale sawmill workers in the Sokoban Wood Village. We have revised the statement in the methods which seeks to portray that we targeted all staff of 181 to provide more clarity. We have also provided an explanation on the adoption of the 25% non-response rate in the revised manuscript. The section in reference now reads as;

***Study population***

*The study population was limited to small-scale sawmill workers at the Sokoban Wood Village in Kumasi.*

***Sample size estimation and sampling procedure***

*The Cochrane formula was used to estimate the sample size for this study [28]:*

$Sample size, SS= \frac{Z^{2}pq}{d^{2}}$*,	Equation 1 [28]*

*where Z is the reliability coefficient of 1.96 at a 95% confidence interval, p is the proportion of sawmill workers that sustained occupational injuries, q is (1 – p) and ‘d’ is the degree of accuracy. Assuming a 78.0% prevalence of occupational injuries among sawmill workers from an earlier study [11], and a 5% degree of accuracy, a sample size of 264 was estimated.*

$$Sample size, S= \frac{{1.96}^{2} x 0.78 x (1-0.78)}{{0.05}^{2}}=264 Equation 1$$

*We adjusted the sample size to 110 for a finite population, N, of 181 (which is the total number of small-scale sawmill workers at the Sokoban Wood Village) using Equation 2. This approach is well documented in the literature [23,29–31]. The 181 is the total number of all workers from the small-scale companies. Knowing this was a finite population, we applied the adjustment for it and got the initial sample size.*

$$Adjusted sample size, Ass= \frac{S}{1+ \frac{S-1}{N}} Equation 2$$

*where Ass is the new sample size, S is the estimated sample size, 264, and N is the finite population. Adjusting by 25% for non-response, a total of 138 sawmill workers were recruited for the study. We used 25% because initial reconnaissance assessments showed it was prudent to anticipate that interviews could span over 2 days because of the volume of work for the workers. On this basis, there was no guarantee a worker who had commenced the interview the previous day would come to work the next day. To overcome these uncertainties, we used the relatively high 25% adjustment factor.*

**Reviewer’s comment:** How was the internal validity and reliability done? Was there any computation for the Cronbach alpha values etc? it’s not enough to say use of experienced staff/members improved validity and reliability – where is the evidence? this validity and reliability section is key and how that is ensured need scientific basis (universal approach) like Cronbach alpha computation/analysis.

**Authors’ response:** The section in reference has been revised to reflect that a pretest was conducted to improve the reliability of the questionnaire. Again, the assessment of occupational injuries and other variables of interest in this study were not assessed with Likert scale responses. Hence, we are unable to use Cronbach’s alpha computation/analysis. We have also acknowledged the questionnaire (tool) as a study limitation because it was not validated and recommended readers to be cautious in the interpretation of the results.

*The questionnaire was not validated; however, it was pre-tested among fifteen (15) small-scale sawmill workers in a different district that was not part of the source population to check to improve its reliability and validity. All the necessary corrections were effected before using it for the final data collection*

*Even though the questionnaire was pretested and fine-tuned following that, the lack of a comprehensive evaluation of validity and reliability is a limitation and the findings ought to be taken with caution.*

We hereby submit our revised manuscript for your consideration.

Sincerely,

**DOUGLAS ANINNG OPOKU**

**CORRESPONDING AUTHOR**
